# High expression of fibroblast activation protein (FAP) predicts poor outcome in high-grade serous ovarian cancer

**DOI:** 10.1186/s12885-020-07541-6

**Published:** 2020-10-27

**Authors:** Min Li, Xue Cheng, Rong Rong, Yan Gao, Xiuwu Tang, Youguo Chen

**Affiliations:** 1 Department of Gynecology & Obstetrics, the First Affiliated Hospital of Soochow University, No.188, Shizi Street, Suzhou, Jiangsu Province, 215006 China; 2 Department of Pathology, Obstetrics and Gynecology Hospital Affiliated to Nanjing Medical University, Nanjing, 210004 China; 3 Department of Pathology, the First Affiliated Hospital with Nanjing Medical University, Nanjing, 210029 China; 4 Institute of Suzhou Biobank, Suzhou Center for Disease Prevention and Control, Suzhou, 215004 China; 5 School of Public Health, Medical College of Soochow University, Suzhou, 215123 China

**Keywords:** Fibroblast activation protein (FAP), The Cancer genome atlas program (TCGA), High-grade serous ovarian cancer (HGSOC), Survival

## Abstract

**Background:**

High-grade serous ovarian cancer (HGSOC) is a fatal form of ovarian cancer. Previous studies indicated some potential biomarkers for clinical evaluation of HGSOC prognosis. However, there is a lack of systematic analysis of different expression genes (DEGs) to screen and detect significant biomarkers of HGSOC.

**Methods:**

TCGA database was conducted to analyze relevant genes expression in HGSOC. Outcomes of candidate genes expression, including overall survival (OS) and progression-free survival (PFS), were calculated by Cox regression analysis for hazard rates (HR). Histopathological investigation of the identified genes was carried out in 151 Chinese HGSOC patients to validate gene expression in different stages of HGSOC.

**Results:**

Of all 57,331 genes that were analyzed, *FAP* was identified as the only novel gene that significantly contributed to both OS and PFS of HGSOC. In addition, *FAP* had a consistent expression profile between carcinoma-paracarcinoma and early-advanced stages of HGSOC. Immunological tests in paraffin section also confirmed that up-regulation of FAP was present in advanced stage HGSOC patients. Prediction of *FAP* network association suggested that FN1 could be a potential downstream gene which further influenced HGSOC survival.

**Conclusions:**

High-level expression of FAP was associated with poor prognosis of HGSOC via FN1 pathway.

## Background

Ovarian cancer is one of the major causes of death in females globally. According to 2018 global cancer statistics, 295,414 new cases and 184,799 deaths were reported [1]. In gynecological oncology, ovarian cancer is less prevalent than breast cancer and cervix cancer, however the death rate of OC is the highest [1]. The most recent 2020 cancer statistics in United States also confirmed that ovarian cancer is the fifth cause of deaths of females (13,940 patients, 5% of total cancer-related death), only trailing by lung & bronchus cancer, breast cancer, colon & rectum cancer, and pancreas cancer [2]. According to the NIH Surveillance, Epidemiology, and End Results program (SEER) survival statistics (2009–2015), 5-year survivorship of ovarian cancer is only 47.6% [3], which remained virtually unchanged since the last decade [4].

Based on the immunohistological variation, serous ovarian cancer is the most common subtype of ovarian cancer, which could be further categorized as high-grade and low-grade neoplasm according to tumor Federation International of Gynecology and Obstetrics (FIGO) grade [5, 6]. High-grade serous ovarian cancer (HGSOC) is the most common, aggressive, and fatal type of ovarian cancer. Almost 30% of patients died within 5 years of diagnosis [7], mainly because of lack of disease-specific symptoms, prominent biomarkers, and effective therapy or targeted drugs [8–10]. Despite sharing some similar histological characters and terminology, high- and low- grade SOCs are now acknowledged as two different neoplasms [11]. In 2011, the Cancer Genome Atlas (TCGA) program published the genomic and transcriptomic data of ovarian serous carcinoma, which summarized specific features of HGSOC such as TP53 mutation, extensive DNA copy variation, BRCA1/BRCA2 inactive mutation, CCNE1 aberrations, and other survival-related preliminary transcriptional signatures [12–15].

Fibroblast activation protein (FAP), a cell-surface serine protease, emerges as an imperative factor in cancer-associated fibroblasts (CAFs), especially relevant to tumor occurrence and progression. Structurally, FAP consists of a cytoplasmic tail, a single transmembrane domain, and an extracellular domain [16]. FAP is rarely expressed in healthy adult tissues. However, FAP is usually highly upregulated during tissue remodeling events, including cancers or cancer-associated fibroblasts (CAFs) [17–20]. In addition, FAP is considered as a potential biomarker in certain tumor diagnosis and progression due to its protumorigenic specificity in both enzymatic and non-enzymatic manners [21–24].

In this study, we aim to identify potential biomarkers of HGSOC survival from TCGA ovarian cancer cohort bioinformatics data. We analyzed gene expression, clinical and/or demographic information, and targeted strategies for potential HGSOC biomarkers. In addition, we validated our findings by immunohistological investigation of tissues in a group of Chinese HGSOC patients. Results suggest that FAP expression could be an effective biomarker for HGSOC survival, which warrants further investigation as potential intervention of HGSOC.

## Methods

### Dynamic protein analysis of TCGA database

Gene expression data (379 cases, Workflow Type: HTSeq-Counts) and clinical information were downloaded by the *TCGAbiolink* package in *R* 3.6.0 (https://www.R-project.org/) from the official TCGA website. After screening the clinical database, 320 patients were included for G3 histologic grade indicating HGSOC. Then, 2 of these patients were excluded because of lack of relevant information. Finally, a total of 318 participants were recruited in our study.

For gene expression analysis, we downloaded the entire 57,331-gene data in serous cystadenocarcinoma from the TCGA RNAseq database. After data cleaning, 19 of 57,331 genes were excluded because of their insufficient expression in total gene expression (expressions of these excluded genes were < 1 copy in all participants). Then RNA expression data were transformed to z-scores in survival analysis.

### Participants recruitment

This study followed the Declaration of Helsinki and was approved by the Institution of Research Ethics Committee of the First Hospital affiliated to Soochow University. All participants were fully aware of all protocols of this study, and signed up written consent forms to authorize the utilization of their tissues and relevant information.

For the validation set of the findings in HGSOC TCGA database, 151 Chinese Han patients were diagnosed and recruited by the First Hospital affiliated to Soochow University, the Nanjing Maternity and Child Health Care Hospital, and the First Hospital affiliated to the Nanjing Medical University, from January 2013 to May 2019. After surgery, patients’ tumor tissues were prepared into the paraffin sections. Their demographic and clinical characteristics were collected as well.

### Immunohistochemistry staining (IHC) and IHC score

IHC was performed on paraffin sections of ovarian cancer tissues to characterize target gene (FAP) expression profile. Detailed steps can be found in our previous study [25]. FAP antibody (#66562) was purchased from CST company to incubate the preparing sections overnight at 4 °C for further staining.

Immunohistochemical staining was performed by using the Boster SABC (rabbit IgG)-POD kit (Wuhan, China) with the recommendation of manufacturer. The above-mentioned FAP antibody was used to incubate the preparing sections overnight at 4 °C, and 3, 3′- diaminobenzidine was taken to dye for scoring, which was evaluated by two independent and qualified pathologists who were blinded to actual clinical outcomes. IHC scoring was then established as follows. Percentage of positive cells and intensity of staining of FAP antibody were first calculated and then divided into these three major categories: ≤3, negative or weak; > 3 and ≤ 6, Moderate; > 6, strong.

### Association network prediction of targeting gene

Prediction of functional protein association networks of candidate genes was performed by STRING version 11.0 (https://string-db.org/) and Genecard version 4.13 (www.genecards.org). In order to increase prediction accuracy, only common predictions present in both websites were included.

### GO and KEGG pathway analysis of host gene

The gene ontology (GO) functional annotation and KEGG pathway analysis of host genes of polymorphisms were carried out by using the package ‘clusterProfiler’ in R (version 4.0.1).

### Statistical analysis

All statistical analyses were calculated in *R* 3.6.0. Overall survival (OS) and progression-free survival (PFS) analyses in SOC patients were conducted by Cox regression and the Kaplan-Meier method. Multivariate Cox analysis was applied to identify potential influence of FAP expression on OS and PFS at different clinical stages of HGSOC. Other relevant demographic and clinical characteristics were compared by Student’s *t* test or Wilcoxon test whenever appropriate between the two groups. For immunohistochemical testing in HGSOC patients, the FAP expression score between Stage I + II and Stage III + IV were compared by Student’s *t*. *P* value < 0.05 was considered as statistical significance in this study.

## Results

### Workflow of gene identification in TCGA database

**Fig. 1:**
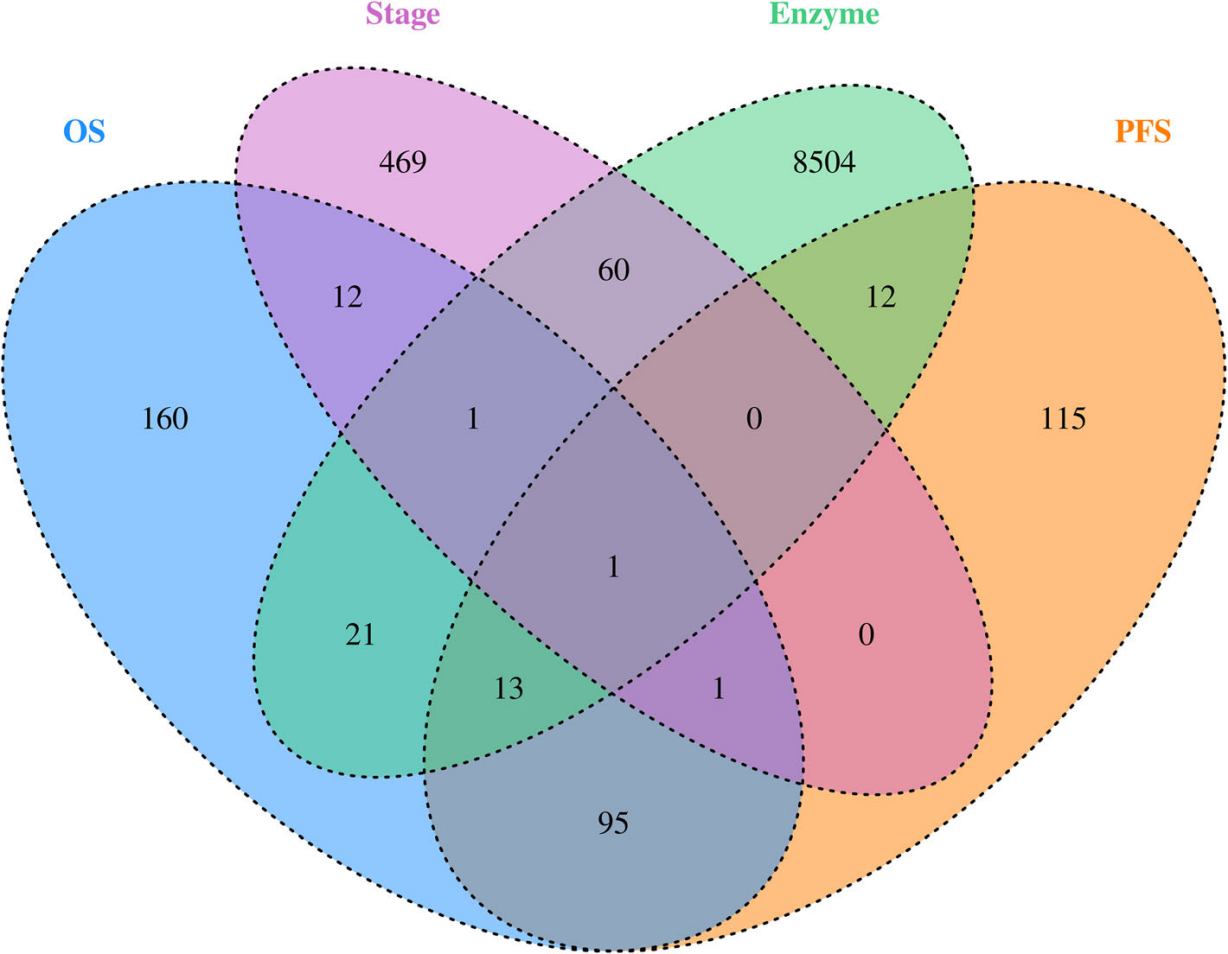
Venn diagram of target genes selection principle

As shown in Fig. 1, there were 304 and 237 genes with different expressions in OS and FPS analysis, respectively. By comparing early stage (Stage I + II) with advanced stage (Stage III + IV) HGSOC, we identified 544 stage-related aberrantly expressed genes in HGSOC patients. After cross-checking OS, FPS and stage-related genes, only *FAP* and *SSC5D* were still present. And, *FAP* was finally included because of its characteristics of typical enzyme-catalyzed activity in uniprot database (www.uniprot.org/) and its potential role in HGSOC patient’s survival (Fig. 1).

### Associations of FAP expression with overall survival (OS) and progression-free survival (PFS)

**Fig. 2:**
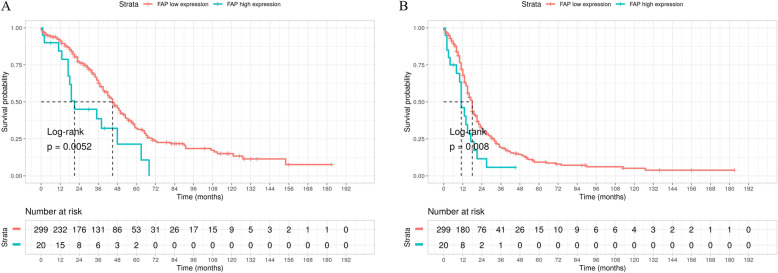
Impact of FAP expression on survival in HGSOC patients in TCGA cohort. **a**. Overall Survival, OS; **b**. Progression Free Survival, PFS

Cox regression analysis based on FAP expression was performed to generate survival curves in OS and PFS. As shown in Fig. 2a, low FAP expression group showed a significant protective effect on HGSOC prognosis in OS (*P* = 0.005). Longitudinally, low FAP expression group had 91.1% survival rate in a period of 12 months, compared with 84.4% in high FAP expression group. Survival rate in 50 months decreased to 31.9% in low FAP group and 21.4% in high group, respectively (Table 1). Results of PFS also showed similar patterns to OS between high and low FAP expression groups (*P* = 0.008, Fig. 2b).

**Table 1:** Significant difference of Predicted gene influenced by FAP in HSOC overall survival COX analysis

Ensembl ID	Gene	β-coefficient	Hazard Rate (HR)	Standard error of coefficient	Z-value	***P***-value
ENSG00000078098	FAP	0.740663	2.097325	0.271298	2.730071964	0.006332
ENSG00000115414	FN1	0.663511	1.941597	0.28008	2.369006093	0.017836
ENSG00000084636	COL16A1	0.916471	2.500451	0.279922	3.274028048	0.00106
ENSG00000130635	COL5A1	0.887919	2.430066	0.289441	3.067700678	0.002157
ENSG00000144810	COL8A1	0.768551	2.15664	0.279769	2.747088883	0.006013
ENSG00000187498	COL4A1	0.608281	1.837271	0.289627	2.100225766	0.035709

### Expression of FAP in HGSOC patients

**Fig. 3:**
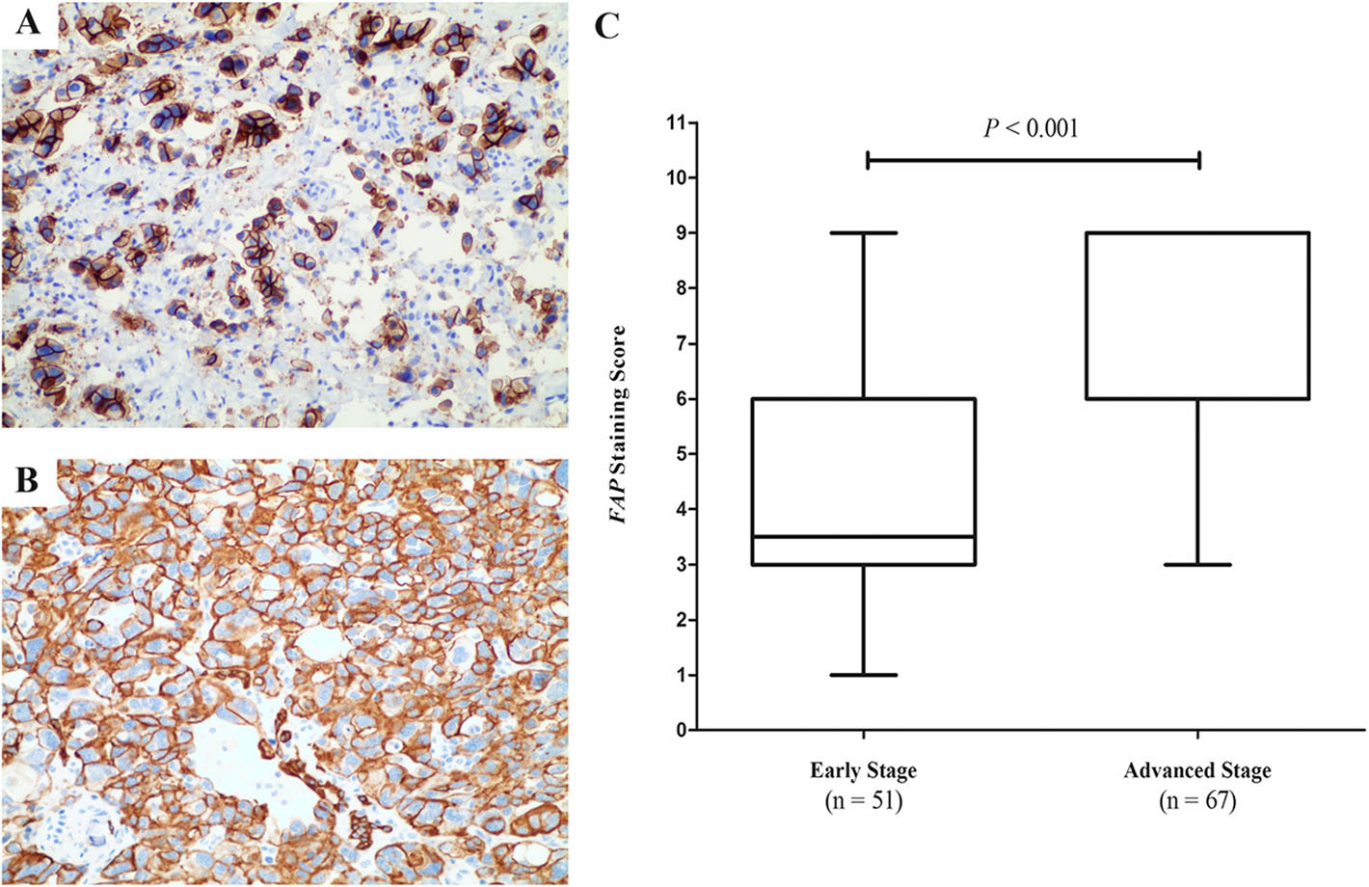
Expression of FAP in HGSOC tissues. **a**. Expression of FAP in early-stage patients’ tissues. **b**. Expression of FAP in advanced-stage patients’ tissues. **c**. Total expression of FAP staining in patients with different stages of HGSOC

Sections of 151 HGSOC patients’ tumor tissues in different stages were stained with FAP-antibody. We identified an increasing trend of FAP expression with respect to severity of cancer stages (Fig. 3). In addition, strong positive FAP-staining generally showed in membranous and cytosolic compartments in HGSOC tissues. Figure 3b revealed the total difference scores between early stage HGSOC patients and advanced patients (*P* = 0.016). Additionally, negative FAP was observed in 33 (21.85%) patients.

### Prediction of network influenced by FAP

**Fig. 4:**
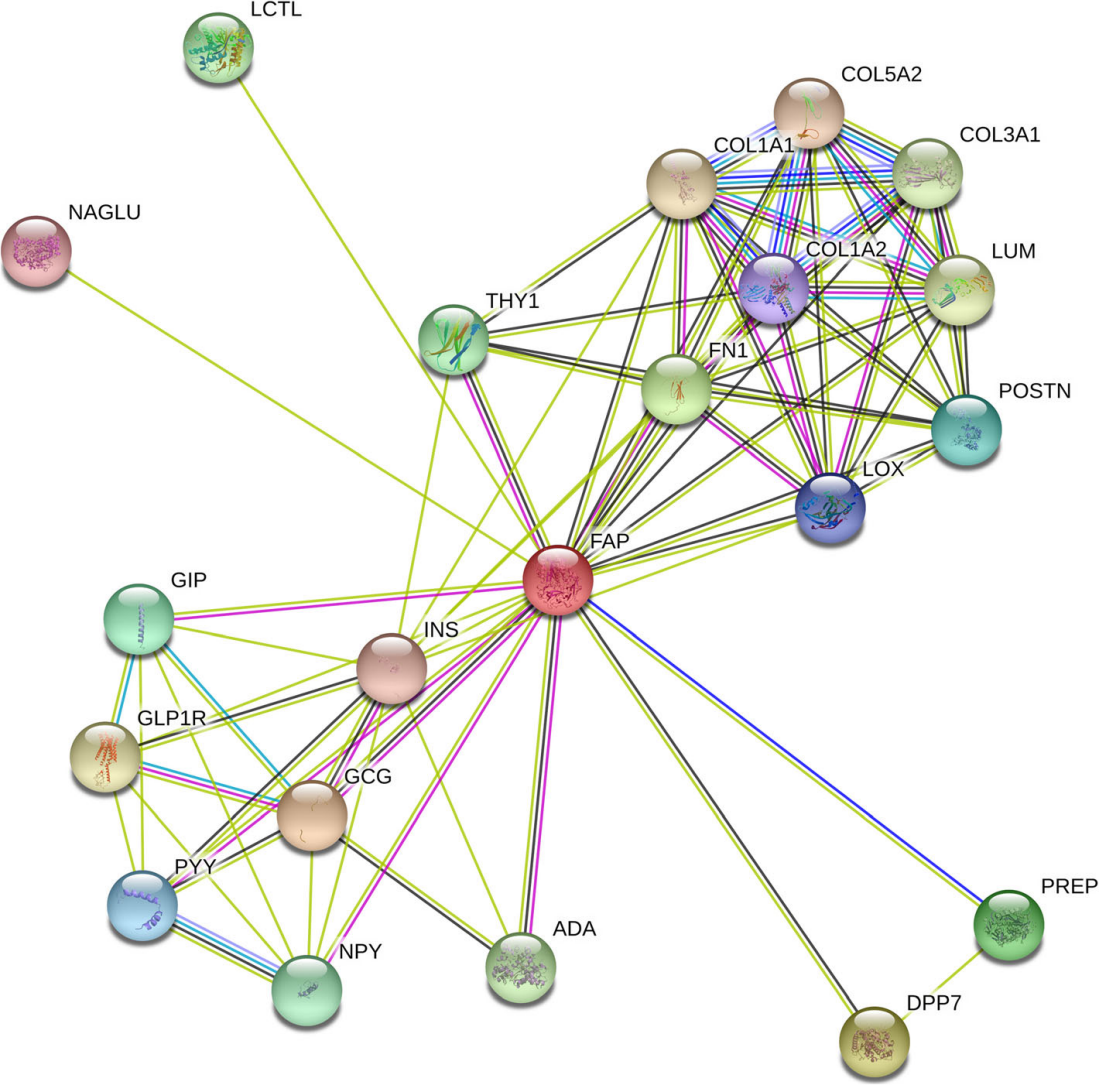
Prediction of FAP influence in gene regulation

Based on STRING and Genecard, prediction of co-influence genes with FAP and their potential regulating effects was shown in Fig. 4. Specifically, fibronetin-1 (FN1), collagen family genes-COL1A1, COL1A2, COL3A1, COL5A2, Thy-1 cell surface antigen (THY1), and insulin (INS) were identified as co-influence genes.

FN1 was the only significant gene based on Cox regression with its hazardous influence on HGSOC (*P* = 0.018, as shown in Table 1). FN1 is demonstrated to over-express in ovarian cancer, which could eventually influence the formation of multicellular aggregate of ovarian cancer cells, migration and invasion of cancer cells, and aggravating platinum-resistance to deactivate chemotherapy. Additionally, epithelial-mesenchymal transition of ovarian cancer is shown to relate to aberrant expression of FN1. Therefore, FN1 and its regulatory factors, including genes, non-coding RNAs and epigenetic regulations, might be valuable candidates for ovarian cancer studies.

Considering the similar function of collagen (COL) family, we extended the search of collagen encoding genes. Additional COL genes that could influence HGSOC included COL16A1 (HR = 2.50, *P* = 0.001 for Cox regression), COL5A1 (HR = 2.43, *P* = 0.002), COL8A1 (HR = 2.16, *P* = 0.006), and COL4A1 (HR = 1.83, *P* = 0.035) (Table 1).

### Bioinformation of FAP in GO and KEGG analysis

**Fig. 5:**
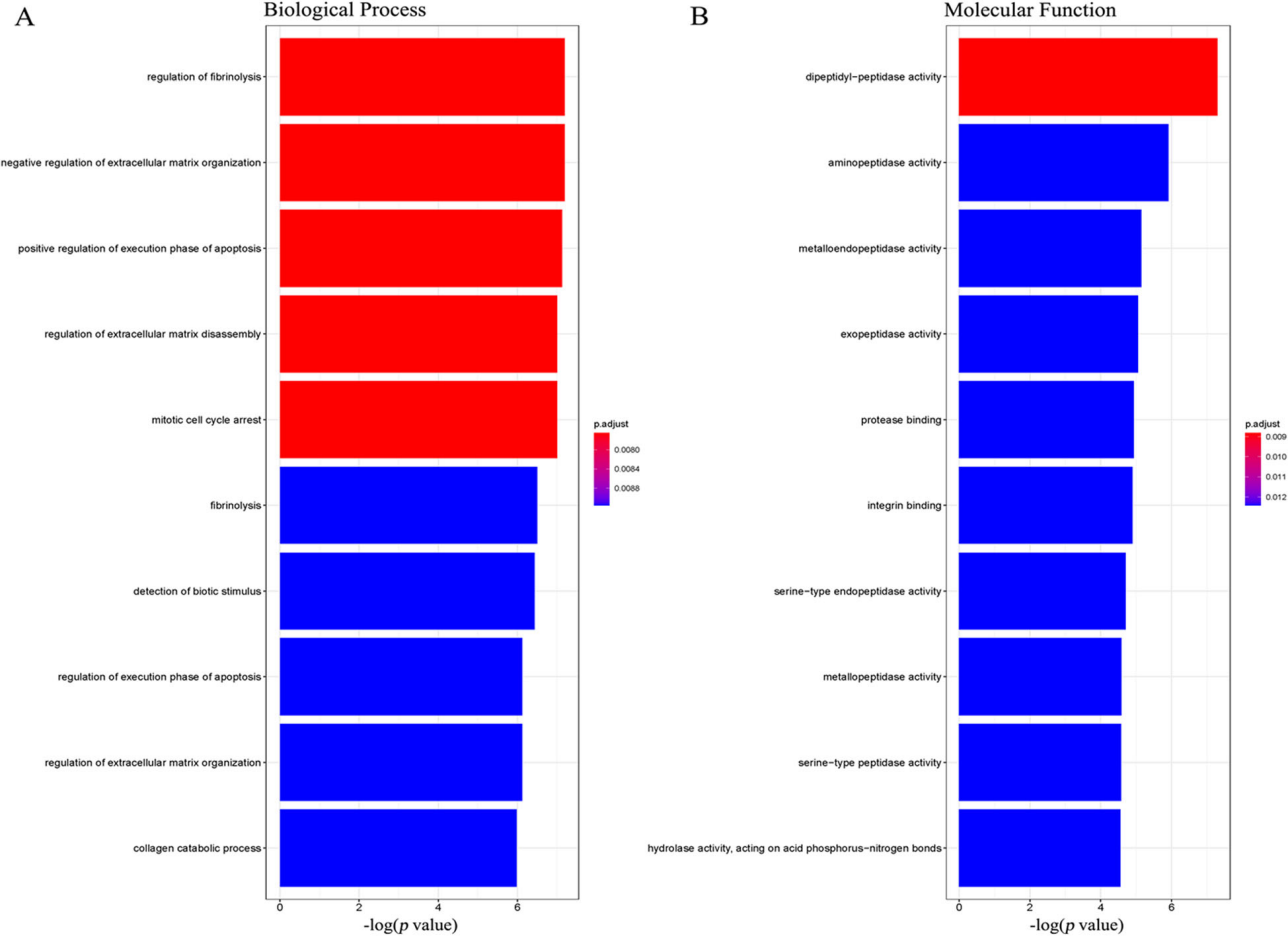
GO annotations of FAP. **a**. Biological Process. **b**. Molecular Function

For gene ontology (GO) analysis of FAP, the top three enriched GO annotations were listed as “regulation of fibrinolysis”, “negative regulation of extracellular matrix organization”, and positive regulation of execution phase of apoptosis” three in Biological Process, while as “dipeptidyl-peptidase activity”, “aminopeptidase activity”, and “metalloendopeptidase activity” in Molecular Function (Fig. 5). On the other hand, however, for KEGG analysis, there is no related available record for FAP in corresponding database.

## Discussion

In this study we extensively searched the TCGA database for HGSOC patients to identify significant biomarkers for HGSOC survival. We constructed a comprehensive summary of differentially expressed genes (DEGs) of HGSOC, especially with regard to patients’ survival, in order to evaluate the effects of gene expression on HGSOC survival through data mining.

FAP is a typical plasma membrane-bound serine protease, which is implicated in matrix digestion and invasion [26, 27]. Overexpression of FAP has been investigated and believed to be associated with prognosis in many diseases, especially in cancers [20, 22, 28]. Some studies have demonstrated that high FAP expression is a negative prognostic factor for epithelial ovarian cancer [29]. Increased expression of FAP is believed to cause recurrence of epithelial ovarian cancer after chemotherapy [29, 30]. Considering that HGSOC is a common histological type of epithelial ovarian cancer, our findings in TCGA database on HGSOC support this finding from a new perspective.

Based on the location of FAP in HGSOC tissue, we suggest that FAP could be a strong positive cell-surface receptor. FAP has high expression in HGSOC patients in both Chinese population (over 60% in our study) and other ethnics (over 50%) [30]. In addition, performance of therapeutic targeting at FAP also suggests its effectiveness for cancers [31]. Therefore, FAP could be a potential biomarker for drug delivery or even direct therapy for HGSOC.

Cancer-associated fibroblasts (CAFs), including FAP, usually participate in extracellular matrix structure remodeling and tumor microarray reconstruction. In this study, we also retrospectively explored the TCGA database and investigated the effects of other CAF members on HGSOC prognosis, such as ACTA2, PDGFRα/β, S100A4, and αSMA. These CAF members are widely adopted as biomarkers of HGSOC. Nevertheless, none of these genes showed significant association with HGSOC survival. This result may be partly due to negative expression of αSMA [30].

We identified *FN1* as the only potential gene regulated by *FAP* in HGSOC. Currently, there is no direct evidence to establish the causal relationship between *FAP* and *FN1*. Nevertheless, in this study, we demonstrated similar eventual clinical outcomes induced by *FAP* and *FN1*, positive correlation between their expression, significance of their role in overall survival of HGSOC, and direct regulating relationship predicted by STRING and Genecard. All these novel findings suggested *FAP* as a novel trigger for *FN1*, at least for HGSOC survival. For *THY1* gene, though it was reported as a putative tumor suppressor of ovarian cancer, our study did not produce the same results as previous ones [32], despite *THY1*’s presence in our prediction of potential *FAP* association networks. It is possible that *THY1* cooperates with *FAP* during HGSOC occurrence but not in prognostic period.

## Conclusion

After extensive data mining of TCGA database, we identified FAP as a significant biomarker for HGSOC survival. FAP overexpression led to worse outcome of HGSOC patients, especially in the advanced clinical stage. *FN1* expression is potentially down regulated by *FAP* and further influences HGSOC survival.

## Data Availability

The main datasets for screening DEGs and analysis during the current study are available from TCGA database. And the corresponding validation IHC data are available from the corresponding authors upon reasonable request.

## Abbreviations

HGSOC: High-grade serous ovarian cancer
DEGs: Different expression genes
OS: Overall survival
PFS: Progression-free survival
HR: Hazard rate
FAP: Fibroblast activation protein alpha
FN1: Fibronectin 1
TCGA: The Cancer Genome Atlas Program
SEER: Surveillance, Epidemiology, and End Results program
FIGO: Federation International of Gynecology and Obstetrics
CAFs: Cancer-associated fibroblasts
IHC: Immunohistochemistry staining
GO: Gene ontology
KEGG: Kyoto Encyclopedia of Genes and Genomes
THY1: Thy-1 cell surface antigen
INS: Insulin
